# VIDEODINING IN OLDER ADULTS AGING IN PLACE: A FEASIBILITY AND ACCEPTABILITY STUDY

**DOI:** 10.1093/geroni/igz038.3523

**Published:** 2019-11-08

**Authors:** Laura K Barre, Sarah Coupal, Tara Young

**Affiliations:** Cornell University, Ithaca, New York, United States

## Abstract

Loneliness and a loss of commensality contribute to the decline in nutritional status observed in older adults. The use of video chatting while dining, i.e. “VideoDining”, provides an opportunity for older adults to eat with another person virtually while dining at home. We tested the acceptability and feasibility of VideoDining in older adults receiving Meals on Wheels (MOW) and explored whether it changed meal intake. Participants were recruited from a rural county in NY and ate their MOW meal while VideoDining with a companion diner at a different location. To assess acceptability, we conducted semi-structured qualitative interviews with each participant and companion diners completed a written survey. The amount of the VideoDining meal consumed was compared to usual intake from three days of food records. 140 MOW clients were contacted,13 agreed to participate and 10 completed the VideoDining experience. Barriers to participation included being uncomfortable with the technology, lack of internet service and illness. Participants were 80% female, 100% white, and all lived alone. Average meal length was 39 minutes and 40% ate more than usual, 30% ate the same, and 30% ate less. Reasons for eating less included being nervous and eating when not their usual mealtime. All participants reported they would VideoDine again and companion diners rated the overall experience a 9.2 out of 10. Older adults are able to VideoDine with a new acquaintance and have a positive experience. Further study is needed to determine if VideoDining can increase dietary intake and decrease loneliness in older adults.

